# Cross With Caution: Antibiotic Cross-Reactivity and Co-Reactivity Patterns in Severe Cutaneous Adverse Reactions

**DOI:** 10.3389/fimmu.2021.601954

**Published:** 2021-02-25

**Authors:** Grace Thompson, Andrew McLean-Tooke, Michaela Lucas

**Affiliations:** ^1^ Queen Elizabeth II Medical Centre, Department of Clinical Immunology, Sir Charles Gairdner Hospital, Pathwest, Nedlands, WA, Australia; ^2^ Medical School, University of Western Australia, Nedlands, WA, Australia

**Keywords:** severe cutaneous adverse drug reactions, antibiotic cross-reactivity, antibiotic co-reactivity, beta-lactam, antibiotics

## Abstract

Current understanding of cross-reactivity in severe cutaneous adverse reactions to beta-lactam antibiotics is limited, thereby making recommendations for future prescribing difficult. The underlying immunopathogenesis of these reactions is not completely understood but involves interactions between small molecule drugs, T cells and HLA molecules. Historically, these reactions were considered to be specific to the inciting antibiotic and therefore likely to have minimal cross-reactivity. We assessed patients presenting with non-SJS/TEN severe cutaneous adverse reactions to a tertiary hospital drug allergy clinic. In our case series cross-reactivity or co-reactivity commonly occurred among the beta-lactam antibiotic class, however further research is required to investigate and understand patterns of cross-reactivity. Based on our experience we provide clinicians with a practical algorithm for testing for cross-reactivity in non-SJS/TEN severe cutaneous adverse reactions.

## Introduction

Severe cutaneous adverse reactions (SCAR’s) are a heterogeneous group of delayed T cell mediated hypersensitivity reactions, which include Stevens-Johnson syndrome (SJS), toxic epidermal necrolysis (TEN), drug reaction with eosinophilia and systemic symptoms (DRESS) and acute generalized exanthematous pustulosis (AGEP) (1). Symmetrical drug-related intertriginous and flexural exanthema (SDRIFE) is another delayed cutaneous exanthema which can be severe. Medications are the most common cause of SCAR’s causing >85% of cases of SCAR’s in adults, of which beta-lactams are frequently implicated (2, 3). Although these conditions are rare, they carry significant morbidity and mortality, particularly if the offending drug is not withdrawn (1). Mortality rates of up to 67% in TEN, 40% in SJS and 10% in DRESS have been reported (4).

Interactions between the culprit drug, human leukocyte antigen (HLA) class I molecules and T cell receptors (TCR) in addition to other factors such as elevated plasma concentrations of the offending drug and viral infectious triggers are all thought to contribute to the immunopathogenesis of all types of SCAR’s (1, 5). The interactions between the TCR, HLA molecule and the offending drug are thought to occur in three possible ways. Firstly, in the hapten/prohapten model a drug binds to a protein that then undergoes antigen processing to generate haptenated-peptides which are recognized as neo-antigens by T cells. Beta-lactam antibiotics have been shown to behave in this manner, as drug modified human serum albumin has been isolated from individuals utilizing piperacillin, penicillin-G and flucloxacillin (6). Furthermore, the sites where drug modification occurs have been mapped using mass spectrometry and synthetic penicilloyl-adduct peptides have been shown to be more potent stimulators of T cells in patients with penicillin hypersensitivity (6). Secondly, the p-i model proposes that small molecule drugs may bind non-covalently to HLA or T cell receptors and directly stimulate T cells (5). Flucloxacillin -mediated delayed hypersensitivity reactions may also act *via* this mechanism, as some flucloxacillin-reacting T cell clones react immediately to flucloxacillin in the presence of antigen presenting cells, which is too rapid for the hapten/prohapten mechanism to occur (7). Lastly, the altered peptide model suggests small molecule drugs can bind non-covalently to the binding cleft of HLA and alter its conformation resulting in presentation of novel peptide ligands which then elicit an immune response (5). To date there is no current evidence for this occurring in beta-lactam hypersensitivity reactions (8).

The resultant characteristic clinical manifestations are then defined by the various effector cells involved. SJS and TEN are considered a continuum of the same disorder, in which CD8+ cytotoxic T cells and NK cells targeting skin keratinocytes can lead to a severe, life threatening exfoliative dermatitis and as a result they are often considered separately from the other SCAR’s. In DRESS syndrome drug specific T cells are thought to mediate perforin/granzyme B and Fas/Fas ligand related cell death leading to the characteristic clinical features of widespread rash, eosinophilia, fever and internal organ involvement, most commonly liver injury (1, 9). Histopathology shows an interface dermatitis with spongiosis and expansion of T regulatory cells and eosinophils in the skin (9). Other factors such as mutations in drug metabolizing enzymes, HLA type as well as herpes viral reactivation likely contribute to T cell expansion and cytokine production (9). The exact role of herpes virus reactivation, particularly HHV6 reactivation in DRESS is controversial. Reactivation can be found in 43%–100% of DRESS cases and therefore it is likely that such reactivation is not essential for the development of DRESS but may be an aggravating factor potentially resulting in perpetuation of the inflammatory response. The mechanisms through which reactivation occurs are not entirely clear but may relate to a relative immunocompromised status which occurs early in DRESS or due to the direct effect of drugs or drug metabolites on HHV-6 replication (9). Viral reactivation may contribute to DRESS through further stimulating T cell expansion and cytokine production and may lead to T cell generation through heterologous immunity. Heterologous immunity may lead to the generation of drug-specific T cells through activation of cross-reactive HHV6 specific effector memory T cells (5). In AGEP drug specific T cells and NK cells are activated in the skin inducing apoptosis of keratinocytes *via* Fas/Fas ligand interactions. Production of cytokines and chemokines such as IL17 and CXCL8 leads to neutrophilic inflammation and formation of pustules which is the clinical hallmark of this condition. Histopathological features include spongiform subcorneal and/or intradermal pustules with oedema of the papillary dermis and a polymorphic perivascular infiltrate can be seen (1). Genetic variants in IL36 receptor antagonist gene have also been identified as a potential susceptibility factor (10). The precise pathophysiology of SDRIFE is unknown although it is thought to involve a type IV delayed-hypersensitivity immune response, as it occurs within a few hours to days following drug exposure. There is evidence of a T cell mediated reaction, with patch testing being positive in up to 50% of patients and delayed intradermal testing being positive in up to 70% of patients (11). While strong pharmacogenomic HLA associations have emerged for certain SCAR syndromes and medications, such as carbamazepine-induced SJS/TEN and HLA-B15:02, there is a lack of information regarding known HLA-associations with beta-lactam-induced SCAR’s (1, 8)

Cross-reactivity can occur between structurally similar medications and is well described in aromatic anti-convulsant related SCAR’s. However, evidence surrounding cross-reactivity in beta-lactam SCAR’s is limited (12). Cross-reactivity in both immediate, IgE-mediated, and benign delayed beta-lactam antibiotic hypersensitivity may be due to either reactivity against the beta-lactam ring or more commonly due to shared identical or similar side chains, most commonly the R1 side chain, and has been reported in up to 31.2% of non-SCAR delayed T cell mediated penicillin allergy (13). Mechanistically T cell mediated reactions were thought to be more specific to an individual drug than IgE-mediated reactions as T cell receptors recognize small peptide fragments, although specific evidence supporting this is sparse (1, 14). El-Ghaiesh *et al.* demonstrated that piperacillin-specific CD4 and CD8 T cell clones from patients with delayed piperacillin hypersensitivity did not proliferate with other beta-lactam antibiotics even those with similar side chains (14) but, it is important to note that none of these clones were isolated from patients with SCAR’s. In a retrospective review of SJS/TEN cases, two patients were inadvertently given the same or class-related antibiotic post-discharge without reported reaction (12).

Understanding cross-reactivity patterns has important clinical implications as currently recommendations for future antibiotic prescribing must involve a careful balance between the risk of precipitating another severe reaction versus restriction of therapeutic options. In this context we sought to determine if cross-reactivity among the beta-lactam class could be demonstrated in a cohort of non-SJS/TEN beta-lactam SCAR’s and if patterns could be elucidated.

## Materials and Methods

All patients presenting to the Sir Charles Gairdner Hospital and Perth Children’s Hospital Immunology clinic with a diagnosis of a beta-lactam antibiotic related non-SJS/TEN SCAR between March 2016 and June 2020 underwent standardized assessment. We receive on average 550–650 adult and 250–300 pediatric drug allergy referrals per year. The majority are for presumed IgE mediated and non-SCAR non-immediate reactions, not all relate to beta lactam antibiotics. As SCAR reactions are also managed by Dermatology, we cannot exclude that we have not been referred all cases. Patients were included if they were deemed to have a clinical diagnosis of a non-SJS/TEN SCAR as made by a specialist immunologist and had either a positive patch or intradermal test to the suspected culprit antibiotic. Patients were excluded if they did not complete assessment. During this period 11 patients were identified with a non-SJS/TEN SCAR secondary to a beta-lactam antibiotic. One patient was excluded as they did not complete testing and one patient was excluded as they were negative on both patch and intradermal testing to all beta-lactam antibiotics.

Our standardized assessment consisted initially of patch testing to the culprit antibiotics. Patch test were applied to the patients back and left in place for 48 h. Results were read using a semi-quantitative score from no reaction, to +, ++, +++ depending on the degree of skin reaction at 48 h, 72 h and 1 week post initial application. Concentrations were based on non-irritating concentrations for patch testing reported in the literature (15, 16). Concentrations used for patch testing included: benzylpencillin 5% and 10%; penicillin VK 1%, 5%, 10%; amoxicillin 5%, 10%, and 25%; ampicillin 5%; flucloxacillin 1%, 5%, and 10%; cephalexin 5% and 10%; ceftriaxone 5% and 10%; cefepime 5%; cephazolin 5%; tazocin 5% and meropenem 5%.

Patients then went on to have intradermal testing (IDT) with delayed readings to beta-lactam antibiotics: if they had a positive patch test to the culprit antibiotic, this antibiotic alone was *typically* excluded on the IDT. Beta-lactam antibiotics included in this panel were: benzylpenicillin 6 mg/ml, Diater^®^ PPL (major determinant) neat, Diater^®^ MDM (minor determinant) neat, amoxicillin 20 mg/ml, ampicillin 20 mg/ml, amoxicillin/clavulanic acid 20 mg/ml, flucloxacillin 2 mg/ml, piperacillin-tazobactam 4.5 mg/ml, cephazolin 1 mg/ml, ceftriaxone 1 mg/ml, cefepime 1 mg/ml, aztreonam 1 mg/ml, and meropenem 2.5 mg/ml (16, 17). Delayed readings were performed at 48 h, 72 h and 1 week (**Figure 2**). The study was approved for conduct by Sir Charles Gairdner Hospital quality improvement committee (GEKO 28972) and Perth Children’s Hospital quality improvement committee (GEKO 26921).

## Results

Nine patients were seen with a confirmed diagnosis of a non-SJS/TEN, beta-lactam related SCAR of which 7 (78%) had evidence of cross-reactivity on our testing. The majority of the patients had DRESS syndrome (7/9) with one patient having AGEP and the other having SDRIFE. The average age was 66 years (11–81 years) with a male to female ratio of 4:5. The average time to testing, taken from first onset of symptoms, was 8 months (1-18 months).

### Case 1

**Figure 1:**
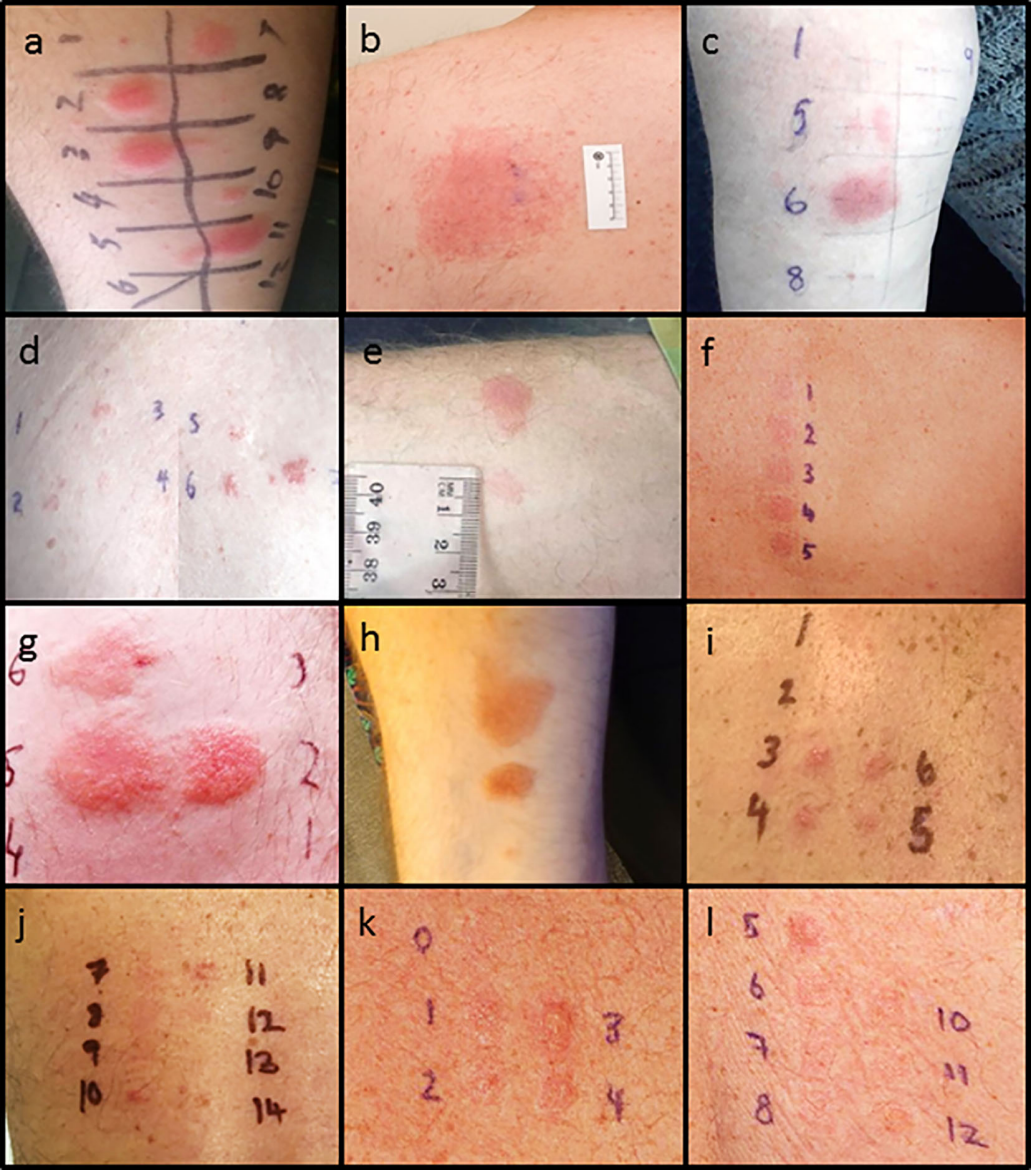
Delayed intradermal and patch test results for cases. **(A)** Case 1 positive delayed intradermal to benzylpenicillin 6 mg/ml (2, 3), flucloxacillin 2 mg/ml (7), piperacillin-tazobactam 4.5 mg/ml (10) and ampicillin 20 mg/ml (11) at 8 h **(B)** Case 1 positive patch test to amoxycillin 10% and 25%s. **(C)** Case 2 positive amoxicillin 20 mg/ml (5) and ampicillin 20 mg/ml (6) **(D)** Case 2 positive patch test to penicillin VK 1%, 5%, 10% (2, 3, 4) and flucloxacillin 1%, 5% and 10% (5, 6, and 7). **(E)** Case 3 positive intradermal test to benzylpenicillin 6 mg/ml (upper) and ampicillin 20 mg/ml (lower). **(F)** Case 3 patch test positive to penicillin VK 10% (2) and benzylpencillin 10,000 IU/g (4). **(G)** Case 4: Positive intradermal test to benzylpenicillin 6 mg/ml (2), ampicillin 20 mg/ml (5), and amoxycillin 20 mg/ml (6) at 72 h. **(H)** Case 4: Positive intradermal test to amoxycillin-clavulanic acid 20 mg/ml (upper), flucloxacillin 2 mg/ml (middle) and equivocal piperacillin-tazobactam 4.5 mg/ml (lower). **(I)** Case 6: Positive patch test to ceftriaxone 10% (3), cefepime 5 and 10% (4, 5), meropenem 5% (6). **(J)** Case 6: Positive patch test to ciprofloxacin 10 and 20% (10, 11). **(K)** Case 7: Positive patch test to benzylpenicillin 10% (1), penicillin VK 10% (2), amoxycillin 10% (3), ampicillin 10% (4). **(L)** Case 7: positive patch test to flucloxacillin 10% (5), cephalexin 10% (6), vancomycin 10% (7), bactrim 10% (8), ceftriaxone 5%(10), cephazolin 5% (11), meropenem 5%(12). Patch test concentrations: benzylpencillin 5% and 10%; penicillin VK 1%, 5%, 10%; amoxicillin 5%, 10%, and 25%; ampicillin 5%; flucloxacillin 1%, 5%, and 10%; cephalexin 5% and 10%; ceftriaxone 5% and 10%; cefepime 5%; cephazolin 5%; tazocin 5% and meropenem 5%. Intradermal test concentrations: Benzylpenicillin 6 mg/ml, Diater^®^ PPL (major determinant) neat, Diater^®^ MDM (minor determinant) neat, amoxicillin 20 mg/ml, ampicillin 20 mg/ml, amoxicillin/clavulanic acid 20 mg/ml, flucloxacillin 2 mg/ml, piperacillin-tazobactam 4.5 mg/ml, cephazolin 1 mg/ml, ceftriaxone 1 mg/ml, cefepime 1 mg/ml, meropenem 2.5 mg/ml, and aztreonam 1 mg/ml.

Thirty-four-year-old male developed AGEP following his second dose of amoxicillin for an upper respiratory tract infection. He developed widespread pustulosis, neutrophilia (10.29x10^9^/L), mild eosinophilia (0.97x10^9^/L) and hepatitis (ALT 104U/L). The rash improved following antibiotic cessation and topical corticosteroids. He had a history of rash to an unknown antibiotic in childhood but had no other exposure to antibiotics since. He had no other significant past medical history. Allergy testing was performed 7 months after his initial reaction. Patch testing to amoxicillin was positive. IDT with delayed readings were positive to benzylpenicillin, flucloxacillin, piperacillin-tazobactam, and ampicillin (**Tables 1** and **2**, **Figure 1**).

**Table 1:** Clinical details of cases.

	1	2	3	4	5	6	7	8	9
Age	34	56	41	31	11	41	63	78	39
Sex	M	F	M	M	F	M	M	F	F
Cormorbidities	Nil	Obesity, HTN, OA	Nil	AR, eczema, food allergy	CF	Nil	BKA	HTN, hyperchol, OA, TIA	AIH
Beta lactam antibiotic implicated	Amx	Flx	Ben Pen	PmPen	Taz	Multiple	Mero	Cef	Taz, Mero
Indication for antibiotic	URTI	Cellulitis	Pneumonia	Tonsilitis	CF	Septic Arthritis	Cellulitis	PJI	Cholangitis
SCAR syndrome	AGEP	DRESS	SDRIFE	DRESS	DRESS	DRESS	DRESS	DRESS	DRESS
Probability score	Naranjo score 6	Regi-SCAR 5	Naranjo score 6	Regi-SCAR 3	Regi-SCAR 3	RegiSCAR 5	Regi-SCAR 5	RegiSCAR 7	RegiSCAR 6
Clinical manifestation	Pustular rash, neutrophilia, hepatitis	Rash, eosin, vomiting/diarrhea, AKI	Erosive flexural rash, hepatitis, eosin	Rash, fever, arthritis, eosin	Rash, fevers, facial swelling eosin, hepatitis	Rash, fevers, LN, eosin, lymphocytosis	Rash, fevers, facial oedema hepatitis eosin	Rash, fevers, eosin, pulmonary infiltrates	Rash, eosin, fevers
Treatment	Top cst	Cst	Cst	Cst	Cst	Cst	Cst	Cst	Cst

HTN, hypertension, OA, osteoarthritis; AR, allergic rhinitis, CF, cystic fibrosis; BKA, bellow knee amputation; hyperchol, hypercholesterolaemia; TIA, transient ischemic attack; URTI, upper respiratory tract infection; PJI, prosthetic joint infection; AKI, acute kidney injury; Amx, Amoxycillin; Flx, flucloxacillin; ben pen, benxylpenicillin; PmPen, phenoxymethylpenicillin; Taz, Piperacillin/tazobactam; Mero, Meropenem; Cef, ceftriaxone; eosin, eosinophilia; LN, lymphadenopathy; top cst, topical corticosteroids; Cst, systemic corticosteroids.

**Table 2:** Patch and intradermal testing results for cases.

Case	Syndrome	Culprit	Time to testing (months)	Beta-lactam antibiotics tolerated post reaction	Patch test to culprit	Delayed intradermal results												
						PPL	MDM	BP	AMX	AMP	AMC	FLU	TZP	CFZ	CRO	FEP	MEM	ATM
1	AGEP	Amoxycillin	7		+^b^	−	−	+ ^a^	NP	+ ^a^	NP	+ ^a^	+ ^a^	−	−	−	−	−
2	DRESS	Flucloxacillin	10		+ ^b^	−	−	−	+ ^a^	+ ^a^	NP	NP	−	−	−	−	NP	NP
3	SDRIFE	Benzylpenicillin	1		+ ^b^	−	−	+ ^a^	−	+ ^a^	NP	−	−	−	−	−	NP	NP
4	DRESS	Phenoxymethyl penicillin	6		+ ^b^	−	−	+ ^a^	+ ^a^	+ ^a^	+ ^a^	+ ^a^	E ^a^	−	−	−	NP	NP
5	DRESS	Piperacillin/tazobactam	16	Amoxycillin with clavulanic acid Cephalexin	+^c^	−	−	−	−	−	NP	−	NP	−	−	−	−	+ ^a^
6	DRESS	Unknown	15		* ^b^	−	−	−	−	−	−	−	−	−	NP	NP	NP	−
7	DRESS	(1)Unknown penicillin (2) Meropenem	6		*^b^	NP	NP	NP	NP	NP	NP	NP	NP	NP	NP	NP	NP	NP
8	DRESS	Ceftriaxone	18	Amoxycillin with clavulanic acid Piperacillin/tazobactam	+^b^	−	−	−	NP	−	−	−	−	NP	NP	−	−	−
9	DRESS	Meropenem	3	Amoxycillin Piperacillin/tazobactam	+^b^	−	−	−	−	−	NP	−	−	−	−	−	NP	−

*Patient 6: Positive to ceftriaxone, cefepime, meropenem, ciprofloxacin. Patient 7: Positive to benzylpenicillin, penicillin VK, amoxicillin, amipicillin, flucloxacillin, cephalexin, ceftriaxone, cephazolin, meropenem, vancomycin and sulfamethoxazole/trimethoprim.

+= Positive, − = Negative, NP = not performed, E = equivocal.

AGEP, acute generalized erythematous pustulosis; DRESS, drug reaction with eosinophilia and systemic symptoms; SDRIFE, symmetrical drug related intreginous flexural exanthema; PPL, Diater PPL; MDM, Diater MDM; BP, benzylpenicillin; AMX, Amoxycillin; AMP, Ampicillin; FLU, flucloxacillin; TZP, piperacillin-tazobactam; CFZ, cephazolin; CRO, ceftriaxone; FEP, cefepime; MEM, meropenem; ATM, aztreonam.

Time to positivity ^a^24 h, ^b^48 h and ^c^72 h.

### Case 2

Fifty-six-year-old female developed DRESS following 24 h of flucloxacillin for cellulitis. She developed vomiting, diarrhea, and acute kidney injury (creatinine 440 Ummol/L). Antibiotics were changed to cephazolin and clindamycin during which time she developed a widespread erythematous exanthema and eosinophilia (1.2x10^9^/L). She was treated with oral prednisolone 50 mg for 1 week followed by 25 mg for a further week in conjunction with oral antihistamines and topical corticosteroids. She had a background history of obesity, hypertension, osteoarthritis and possible anaphylaxis to contrast. Allergy testing was performed 10 months following her initial reaction. Patch testing was positive to penicillin VK and flucloxacillin. IDT with delayed readings were positive to amoxicillin and ampicillin (**Tables 1** and **2**, **Figure 1**).

### Case 3

Forty-one-year-old male developed SDRIFE following 24 h of benzylpenicillin and azithromycin for treatment of pneumonia. He developed severe erythema with skin erosion in his flexures, hepatitis (ALT 105 U/L), and eosinophilia (1.47x10^9^/L). He was treated with corticosteroids. He had no significant past medical history. Allergy testing was performed one month following his initial reaction. Patch testing was positive to penicillin VK and benzylpenicillin but negative to azithromycin. IDT with delayed readings were positive to benzylpenicillin and ampicillin (**Tables 1** and **2**, **Figure 1**). An outpatient supervised oral challenge was planned to azithromycin but unfortunately the patient did not attend.

### Case 4

Thirty-one-year-old male developed DRESS on day six of phenoxymethylpenicillin for treatment of tonsillitis. He developed a diffuse maculopapular rash, fever, arthritis and eosinophilia (0.8x10^9^/L). He was treated with oral corticosteroids. He had a background history of atopic disease with anaphylaxis to sunflower seeds, allergic rhinitis, and mild eczema. Allergy testing was performed 6 months after his initial reaction. Patch testing was positive to phenoxymethylpenicillin. IDT with delayed readings was positive to benzylpenicillin, amoxicillin, ampicillin, amoxicillin/clavulanic acid, flucloxacillin and equivocal to piperacillin-tazobactam (**Tables 1** and **2**, **Figure 1**).

### Case 5

Eleven-year-old female developed DRESS on day 15 of intravenous piperacillin/tazobactam for an infective exacerbation of cystic fibrosis. She developed fevers, maculopapular rash, facial swelling, eosinophilia (0.81x10^9^/L) and hepatitis (ALT 234 U/L). She was inadvertently subsequently prescribed amoxicillin/clavulanate and cephalexin which she tolerated without reaction. Allergy testing was performed 16 months following the initial reaction. Patch testing to piperacillin/tazobactam was positive. IDT with delayed readings was positive to aztreonam but not performed against piperacillin/tazobactam (**Tables 1** and **2**).

### Case 6

Fourty-one-year-old male developed DRESS in the setting of multiple antibiotics including cefepime, meropenem, ciprofloxacin, vancomycin, rifampicin, clindamycin, flucloxacillin and cephalexin given for treatment of left knee septic arthritis following an elective arthroscopy and meniscal repair. He developed fevers, rash, lymphadenopathy, eosinophilia (1.0 x 10^9^/L) and lymphocytosis (10.7 x 10^9^/L). He required a prolonged course of oral corticosteroids initially 75 mg for 4 days, then 50 mg, followed by a weaning course down to 15 mg over 6 weeks, however on reduction of steroids bellow 15 mg he had recurrence of rash, requiring a slower steroid taper over the subsequent 4 months. While still on 1mg of prednisolone he was treated with cephalexin for a finger laceration and within 3 days of therapy developed fevers and rash. He was treated with a single dose of IV hydrocortisone 200mg and his symptoms resolved. Patch testing 6 months later revealed positive results to ceftriaxone, cefepime, meropenem and ciprofloxacin at 48 h (**Tables 1** and **2**, **Figure 1**).

### Case 7

Sixty-three-year-old male with a background of traumatic below knee amputation developed DRESS syndrome characterized by periorbital oedema, maculopapular rash, eosinophilia (0.69 x10^9^/L) and hepatitis (ALT 347 U/ml) 1 week after commencement of meropenem and vancomycin for treatment of cellulitis. This is on a background of a likely SCAR occurring in 1976 characterized by fevers, erythrodermic skin rash with desquamation and collapse six weeks into antibiotic therapy with a sulphonamide and an unknown penicillin antibiotic. Because of concerns about his historical reaction potentially being SJS/TEN we undertook patch testing to a broad panel of beta-lactam antibiotics, 6 months after the most recent reaction and did not perform IDT with delayed readings. This was positive to benzylpenicillin, penicillin VK, amoxicillin, amipicillin, flucloxacillin, cephalexin, ceftriaxone, cephazolin, meropenem, vancomycin and sulfamethoxazole/trimethoprim at 48 h (**Tables 1** and **2**, **Figure 1**).

### Case 8

Seventy-eight-year-old female developed DRESS syndrome characterized by rash, fevers, eosinophilia (peak 5.9x10^9^/L) four weeks into a course of ceftriaxone and ciprofloxacin for a prosthetic hip joint infection. She also reported dyspnoea with pulmonary infiltrates detected on a CT chest. Her BNP was mildly elevated at 250 and an echocardiogram was normal. She initially responded well to oral corticosteroids but had recurrence of symptoms on multiple attempts at steroid weaning requiring addition of mycophenolate. She had a past medical history of hypertension, hypercholesterolaemia, osteoarthritis and a transient ischaemic attack. Patch testing 18 months later was positive to ceftriaxone. The patient subsequently tolerated amoxicillin with clavulanic acid and piperacillin/tazobactam as well as a ciprofloxacin challenge (**Tables 1** and **2**).

### Case 9

Thirty-nine-year-old female with a background of autoimmune liver disease developed DRESS characterized by rash, fevers and eosinophilia (3.2x10^9^/L) after treatment with multiple antibiotics including tazocin (piperacillin/tazobactam), ciprofloxacin, vancomycin and meropenem for cholangitis. She had been on treatment with azathioprine but this was ceased during this same admission as it was deemed to be ineffective due to progressive liver disease. She responded to treatment with oral prednisolone 50mg which was tapered and ceased over 2 months. Patch testing 3 months later was positive to meropenem. IDT with delayed readings was negative to other penicillin and cephalosporin antibiotics and she subsequently tolerated oral challenges to amoxicillin and ciprofloxacin and a course of piperacillin/tazobactam (**Tables 1** and **2**).

## Discussion

We describe a cohort of nine patients with non-SJS/TEN SCAR in which we found evidence of cross-reactivity in 75% in which the patterns of cross-reactivity seen were not predictable based on reactivity to the beta-lactam ring or the R1 side chain. The mechanisms of cross-reactivity in beta-lactam allergies include reactivity to the common beta-lactam ring, which is rare in IgE mediated allergy and absent in those with T cell mediated allergy (18) or more commonly due to structural similarities between side chain structures, most frequently the R1 side chain. Cross-reactivity between penicillins and cephalosporins in low-risk delayed T cell mediated reactions has been found to occur in 2.8–31.2% of patients (13, 19), most commonly among the aminocephalosporins, but there is limited literature addressing cross-reactivity in beta-lactam SCAR’s specifically. Cross reactivity between penicillins and carbapenems is less than 1% and has is thought to be absent with aztreonam (18).

Our cross-reactivity rate of 75% is higher than what has been described to date in the literature. Romano et al. described a cohort of 214 patients with non-immediate reactions to aminopenicillins, which included eight patients with a SCAR, 5 with TEN and 3 with AGEP. Of those with a non-SJS/TEN SCAR 66.6% (2/3) were found to have either a positive patch or delayed IDT to at least one aminocephalosporin (13). More recently Berot et al. described 56 patients with delayed beta-lactam allergies including 26 patients with non-SJS/TEN SCAR’s. Of these patients, 30% (1/3 DRESS cases; 8/23 AGEP cases) had evidence of cross reactivity on patch testing (20).

In our patients with evidence of cross-reactivity on testing, four patients were positive to multiple penicillins without positivity to cephalosporins, two patients were positive to multiple penicillin and cephalosporin antibiotics as well as meropenem and non-beta-lactam antibiotics and one patient was unusually positive only to Tazocin and aztreonam and had tolerated other beta-lactam antibiotics.

Cases 1–4 had positive testing against multiple penicillins without associated positivity to cephalosporins, suggesting a penicillin class effect. This has been described in the literature before, including in beta-lactam SCAR’s. Watts et al. described a patient with benzylpenicillin DRESS who had evidence of cross-reactivity to amoxicillin on patch and delayed IDT but tolerated cephalexin (21). The mechanism responsible for this pattern of cross-reactivity among the penicillin class is not understood, but may be due to more complex antigen structures following molecular processing, and protein folding during antigen presentation or may be due to coexisting sensitivities to different beta-lactam antibiotics (22). The majority of non-SJS/TEN SCAR patients with cross-reactivity in the Berot et al. study had initially reacted to amoxicillin and then had positive penicillin M and Penicillin G/V patch tests (20). In the Romano et al. cohort the cross-reactivity patterns in the two non-SJS/TEN and two TEN SCAR patients appeared to occur exclusively to aminopenicillins and therefore could be explained by the shared R1 side chain (13). Interestingly based on our testing, none of our cases of cross-reactivity appeared to be due to the R1 side chain.

Case 5 in our study had demonstrable positivity to aztreonam following DRESS secondary to piperacillin/tazobactam, despite the patient subsequently tolerating other beta-lactams including amoxicillin with clavulanic acid and cephalexin. It is unknown whether this result represents a true allergy to aztreonam or a false positive intradermal test as reactivity to aztreonam in patients with delayed penicillin allergy has been thought to be close to zero, although this has only been examined *via* patch and skin testing in eight patients with beta-lactam SCAR’s (13, 22). Another unrelated co-existing sensitisation to aztreonam may be an alternative explanation for this finding.

Multiple drug reactivity (MDR) or co-sensitisation/reactivity is another possible explanation for our findings. MDR is described in DRESS syndrome where multiple positive patch tests are detected to chemically unrelated drugs (23). This phenomenon is very uncommon in other types of cutaneous adverse drug reactions (0.3%) but can occur in up to 18% of DRESS cases (23). This may best explain the results in case 6, in which positivity was found to both a 3^rd^ and a 4^th^ generation cephalosporin, meropenem and ciprofloxacin and in case 7 in which positivity was found to multiple penicillins, 1^st^ and 3^rd^ generation cephalosporins and meropenem as well as vancomycin and sulfamethoxazole/trimethoprim. The underlying pathogenesis of MDR is unknown but a potential explanation is that the enhanced stimulation of the immune response from co-stimulation by viral reactivation and/or the initial drug stimulation could lead to generation of an immune response to another drug-protein conjugate (23).

**Figure 2:**
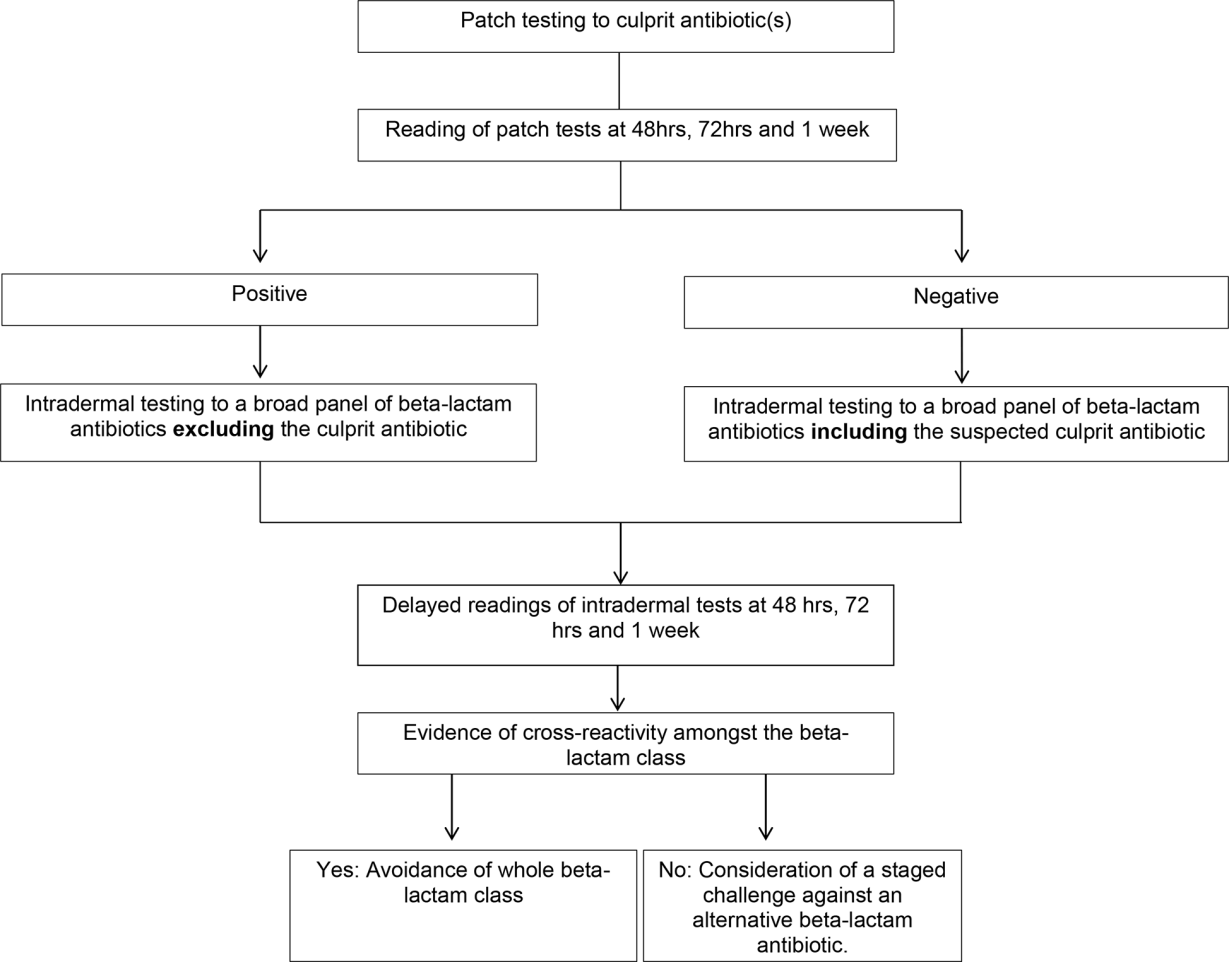
Algorithm for skin testing in non- Stevens-Johnson syndrome (SJS)/toxic epidermal necrolysis (TEN) beta-lactam severe cutaneous adverse reactions (SCAR’s).

Diagnostic testing for drug causality and cross-reactivity is difficult in SCAR’s due to the low sensitivity of testing, the multitude of drugs often implicated and the risk of precipitating a reaction (1). Testing options include a combination of patch and IDT with delayed readings (24). Our approach to testing involves patch testing against the culprit antibiotics in all SCAR’s. In non-SJS/TEN beta-lactam SCAR’s this is followed by IDT with delayed readings at 48 h, 72 h and 1 week, to a broad panel of beta-lactam antibiotics. If the culprit antibiotic is positive on patch testing this is then omitted from the IDT panel. If there is evidence of cross-reactivity on skin testing then avoidance of the whole beta-lactam class is justified. In those cases without evidence of cross-reactivity a graded challenge to an alternative, clinically relevant, oral beta-lactam antibiotic can be considered (**Figure 2**). We typically give 100^th^ of a standard dose, followed by a 10^th^ of a standard dose and then a full dose at one weekly intervals. Locally this approach has been applied at two of the three tertiary hospitals in Western Australia that offer drug allergy testing and we have found this approach to be safe, with all of our cases tolerating skin testing and oral challenges when performed. This is in keeping with the literature that IDT with delayed readings is safe and increases diagnostic sensitivity when patch testing is negative in non-SJS/TEN beta-lactam SCARs (2, 23). This particular algorithm has not been published previously in the literature but is in line with the current European Academy of Allergy and Clinical Immunology (EAACI) guidelines which recommend patch testing as the first line of testing in patients with SCAR’s and proceeding to IDT if PT is negative (22).

Furthermore, we were able to identify a high rate of cross-reactivity to a range of beta-lactam antibiotics on intradermal testing. Our cases highlight that the patterns of cross-reactivity seen in beta-lactam SCAR’s are not always predictable based on reactivity to the beta-lactam ring or to a side chain, and therefore we believe a standardized approach to testing against a wide variety of beta-lactam antibiotics including aztreonam is justified. This approach differs to that of Berot et al. in which patch testing was performed against a panel of penicillin and cephalosporin antibiotics and IDT was only performed if the patch test was negative. As a result this study did not find IDT with delayed readings to be of added diagnostic value (20).

Our study included patients with SCAR’s to a range of beta-lactam antibiotics which is in contrast to Romano’s and Berot’s studies in which 97% and 82.1% of the patients included had previously reacted to an aminopenicillin (13, 20). Finally, in these previous cohort studies they were comprised predominantly of benign delayed drug reactions with a small number of SCAR patients included, making it difficult to assess results on the SCAR patients separately.

There are several limitations to our study. Firstly, none of our cases with evidence of cross-reactivity on skin testing underwent oral challenges, as it is contraindicated, and therefore the true clinical cross-reactivity remains unconfirmed by provocation. Secondly, we are reporting on findings from a small case series which is a direct result of the rarity of these conditions. Finally, we had a relative predominance of DRESS cases in our case series; this is in keeping with the known prevalence of DRESS compared with other SCAR’s in the literature, but may have influenced our results.

Despite these limitations our case series highlights that cross-reactivity or co-reactivity does occur among non-SJS/TEN beta-lactam SCAR’s and potentially may occur more commonly than previously described. Furthermore, the patterns of cross-reactivity we observed were most commonly that of multiple penicillins being positive without cephalosporins or that of multiple drug reactivity or co-reactivity. Interestingly we did not observe cross-reactivity due to the R1 side chain in our cohort which is thought to be the most common cause of cross-reactivity in both IgE and T cell mediated allergy (22). Given the current lack of evidence and understanding around cross-reactivity patterns in beta-lactam SCAR’s a standardized approach to assessment is required. Further research in larger cohorts to better understand the underlying pathophysiology of beta-lactam SCAR’s is also critical to determining cross-reactivity patterns to allow for safe but avoiding unnecessarily restrictive prescribing.

## Data Availability Statement

The raw data supporting the conclusions of this article will be made available by the authors, without undue reservation.

## Ethics Statement

The study was approved for conduct by Sir Charles Gairdner Hospital quality improvement committee (GEKO 28972) and Perth Childrens Hospital quality improvement committee (GEKO 26921). Written informed was obtained from the individual(s) for the publication of any potentially identifiable images in this article.

## Author Contributions

GT - data collection and analysis, wrote paper. AM-T - data contribution and paper review. ML - study design and conception, data contribution and paper review. All authors contributed to the article and approved the submitted version.

## Conflict of Interest

The authors declare that the research was conducted in the absence of any commercial or financial relationships that could be construed as a potential conflict of interest.
